# Coastal Evolution in a Mediterranean Microtidal Zone: Mid to Late Holocene Natural Dynamics and Human Management of the Castelló Lagoon, NE Spain

**DOI:** 10.1371/journal.pone.0155446

**Published:** 2016-05-13

**Authors:** Ana Ejarque, Ramon Julià, Jane M. Reed, Francesc Mesquita-Joanes, Javier Marco-Barba, Santiago Riera

**Affiliations:** 1 CNRS, UMR 6042, GEOLAB, 4 rue Ledru, F-63057 Clermont-Ferrand cedex 1, France; 2 Université Clermont Auvergne, Université Blaise Pascal, GEOLAB, BP 10448, F-63000 Clermont-Ferrand, France; 3 Seminary of Prehistoric Studies and Research, Department of Prehistory, Ancient History and Archaeology, University of Barcelona, 08001, Barcelona, Spain; 4 Department of Geography, Environment and Earth Sciences, University of Hull, Cottingham Road, Hull, HU6 7RX, United Kingdom; 5 Cavanilles” Institute of Biodiversity and Evolutionary Biology, University of València, Av. Dr. Moliner, 50, E-46100, Burjassot, Spain; University of Aveiro, PORTUGAL

## Abstract

We present a palaeoenvironmental study of the Castelló lagoon (NE Spain), an important archive for understanding long-term interactions between dynamic littoral ecosystems and human management. Combining geochemistry, mineralogy, ostracods, diatoms, pollen, non-pollen palynomorphs, charcoal and archaeo-historical datasets we reconstruct: 1) the transition of the lagoon from a marine to a marginal environment between ~3150 cal BC to the 17^th^ century AD; 2) fluctuations in salinity; and 3) natural and anthropogenic forces contributing to these changes. From the Late Neolithic to the Medieval period the lagoon ecosystem was driven by changing marine influence and the land was mainly exploited for grazing, with little evidence for impact on the natural woodland. Land-use exploitation adapted to natural coastal dynamics, with maximum marine flooding hampering agropastoral activities between ~1550 and ~150 cal BC. In contrast, societies actively controlled the lagoon dynamics and become a major agent of landscape transformation after the Medieval period. The removal of littoral woodlands after the 8^th^ century was followed by the expansion of agrarian and industrial activities. Regional mining and smelting activities polluted the lagoon with heavy metals from the ~11^th^ century onwards. The expansion of the milling industry and of agricultural lands led to the channelization of the river Muga into the lagoon after ~1250 cal AD. This caused its transformation into a freshwater lake, increased nutrient load, and the infilling and drainage of a great part of the lagoon. By tracking the shift towards an anthropogenically-controlled system around ~750 yr ago, this study points out Mediterranean lagoons as ancient and heavily-modified systems, with anthropogenic impacts and controls covering multi-centennial and even millennial timescales. Finally, we contributed to the future construction of reliable seashell-based chronologies in NE Spain by calibrating the Banyuls-sur-Mer ΔR offset with ceramic imports from the *Emporiae* archaeological site.

## Introduction

Coastal lagoons are shallow basins connected to the sea by one or more inlets between barrier islands. They form in gently sloping coasts where sea level is rising relative to the shoreline. The relationships between tidal regime, size of the inlets, freshwater inflow, precipitation/evaporation ratios and basin morphology exert a strong influence on water exchange with the adjacent sea, internal circulation, suspended sediment transport and water salinity of the lagoon [1, 2]. These characteristics make coastal lagoons unique aquatic ecosystems, which are characterized by high sensitivity to natural and anthropogenic forcing [3, 4].

Mediterranean lagoons are highly productive ecosystems and since prehistoric times have constituted a vital natural resource for human communities. The range of ecosystem services provided is large, including food production, both within (e.g. shellfish, birds, fish) and around lagoons (e.g. cropping, pasturelands), and industrial exploitation such as salt works, soap and commercial fisheries [5, 6]. Their function as centres of human population benefits also from the proximity of lagoons to river deltas, ensuring freshwater supply. Millennia of coupled climatic-environmental and human exploitation for agriculture, industry and urban development have deeply impacted lagoonal landscapes, contributing to the contraction and loss of littoral wetlands [5].

Coastal lagoons provide invaluable sedimentary archives by which to reconstruct the long-term evolution of the coastal zone, with potential to disentangle the relative influence of climate change, sea-level change and human impact [7, 8]. This knowledge can contribute significantly to predictions of future coastline response to anthropogenic and natural forcing, and to development of sustainable management strategies for the conservation of endangered natural and cultural resources in coastal areas [9]. Geomorphological and palaeoecological research on Mediterranean lagoons has expanded considerably over the last decade. Studies performed in Greece [10, 11], Italy [12, 13]; France [8, 14, 15], and south-eastern Spain [16, 17] have unraveled natural and anthropogenic triggers in the Holocene evolution of coastal landscapes, underlining the intense human exploitation of lagoonal areas from the Neolithic to the present day. However, there are few detailed and well-dated studies of lagoonal ecosystems in NE Spain integrating palaeoecological and archaeo-historical datasets.

**Fig 1:**
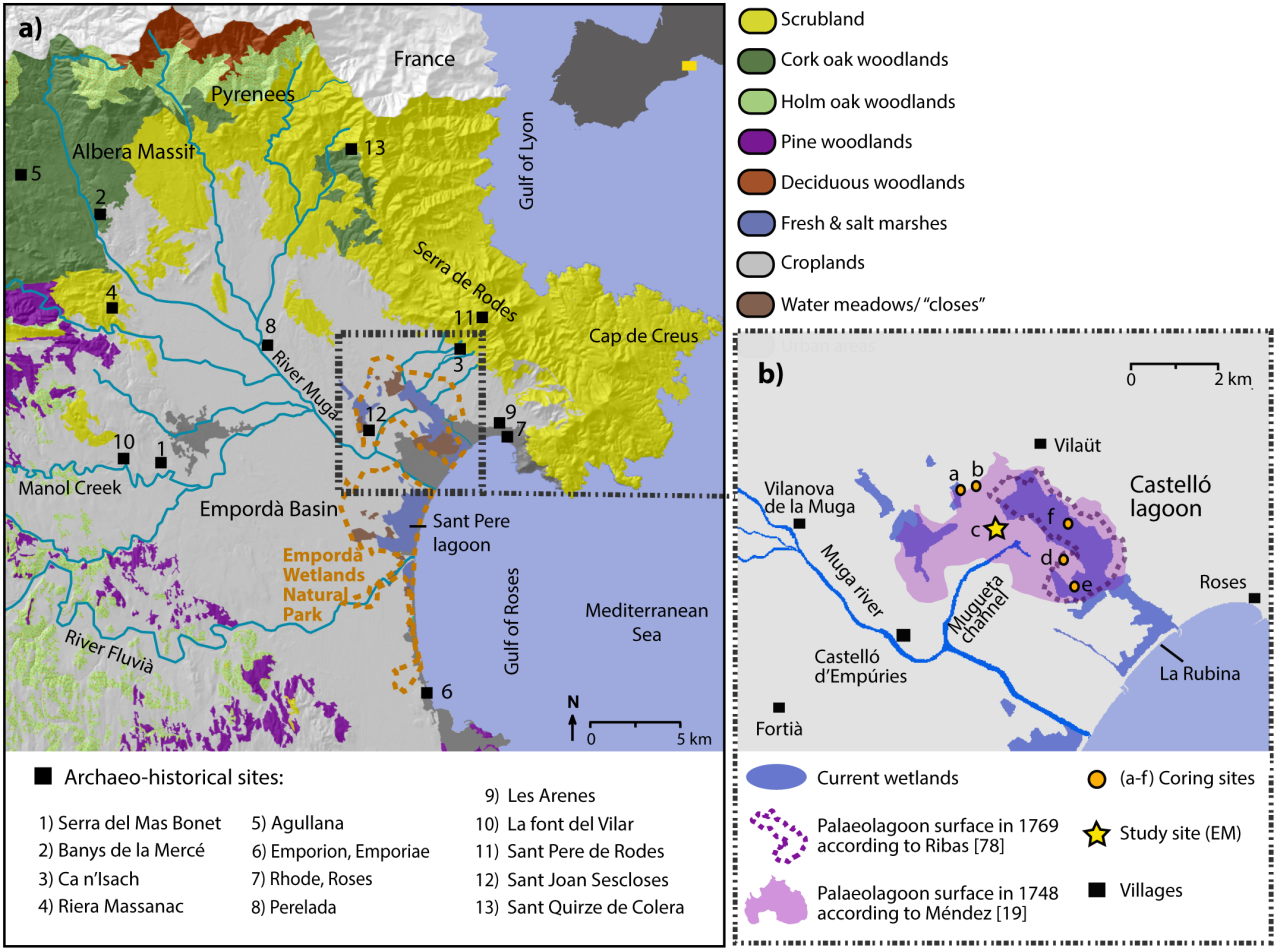
Maps showing the location of the study area. (A) Location of the Alt Empordà and the Castellò lagoon in NE Spain with main vegetation types and archaeological sites mentioned in the text. (B) Detailed location of the EM core site in the EWNP, together with additional coring sites performed in the Castelló lagoon. The figure also shows the quick infilling process of the lagoon in the 2^nd^ half of the 18^th^ century as depicted in ancient maps.

The Empordà Basin (Fig 1) accounts for one of the last remnants of coastal wetlands to survive in the Mediterranean coast of Northern Spain. Coastal lagoons used to be more extensive in the Empordà basin than at present since many have now desiccated either naturally or through land management practices [18]. According to 18^th^ century documentary data and maps, two extensive lagoons existed in the northern Empordà Basin comprising the Sant Pere in the south, and the Castelló lagoon in the north. The latter is occupied by ephemeral and shallow water bodies scattered over an approximate surface area of 350 ha, reflecting its almost total disappearance compared to its former extent of ~1000 ha as depicted in mid-18^th^ century maps [19] (Fig 1B). The Empordà Wetlands Natural Park (EWNP) was established in 1983 to protect what remained of the lagoon complex, included in 1993 within the RAMSAR list of wetlands of international importance. Despite its significance, only one palaeoenvironmental study has been performed in the Castelló lagoon [20, 21], and reversed ^14^C dates compromised the reliability of the age model and the interpretation of the sequence.

In this paper, we present a palaeoenvironmental study of the Castelló lagoon in order to fill a gap in current research for this area by reconstructing the lagoon’s palaeoenvironmental dynamics and its complex history of human exploitation. Palaeoenvironmental changes recorded in the sedimentary sequence could inform on sea-level changes, the morphological configuration of the coastline, the process of lake infilling and the drainage of the basin. To this end, we use a range of proxy indicators comprising mineralogy, pollen, non-pollen palynomorphs, charcoal, diatoms and ostracods. These have been coupled with available archaeological and historical data-sets in order to trace the impact of land-use changes on the Empordà wetlands and surrounding landscapes during the last 5000 years. A major focus is also to resolve for this region one of the most common limitations of palaeoenvironmental research in coastal lagoons, namely the construction of reliable chronologies. This is due to the marine reservoir effect of seashells, the most abundant and well-preserved remains used for ^14^C dating in lagoon sediments [22]. To this end we calibrate the nearest ΔR value available for this region by comparing radiocarbon dates of marine shells with dates derived from stratigraphically-associated ceramic imports in nearby archaeological levels.

### Study area

### Geographic setting

Our palaeoenvironmental study was carried out in the central area of the ancient Castelló lagoon. This is located at the northeast border of the Empordà basin, at the southern foothills of the Pyrenees, NE Iberian Peninsula (Fig 1A). The lagoon complex formed in a depressed area overlying granitic and metamorphic rocks of the Serra de Rodes and Neogene sedimentary deposits of the Empordà basin. The Castelló lagoon is fed by the fluvial discharges of the Muga river, and it is separated from the sea by a sandy coastal barrier ~4 km wide known as La Rubina, where various beach ridges, backshore depressions, lagoon inlets and outlets are observed (Fig 1B). La Rubina acts as a porous barrier to seawater inflow during storm events, and as an outflow drainage area when the lagoon overflows due to floods or irrigation input.

### Regional climate and vegetation

The study region is characterized by a maritime Mediterranean climate. Climatic series from the Castelló d’Empύries climate station [23] record a mean annual rainfall of 712 mm, with an average maximum of 303 mm during autumn and an average minimum of 78 mm during summer. Mean annual temperature ranges from 9.3°C in February to 23.7°C in July and August. The zone is exposed to the Tramontane, NNW dry and fresh winds mainly blowing during the winter months and often reaching speeds of >100 km/h. The zone is also exposed to occasional easterly gale-force Levantine winds, which can cause storm surges and coastal flooding during autumn months. The storms can cause high-energy waves up to >1 m high in this typically Mediterranean microtidal area where the average tidal range is only 0.15 m. The combination of wind patterns and northerly sea currents from the Gulf of Lyon exert a strong influence on coastal dynamics [24].

The vegetation of the littoral plain comprises a mosaic of ecosystems including freshwater and salt marshes, lakes, flowing streams, irrigated channels and the characteristic “closes”, flooded hay meadows limited by drainage canals and bordered with riparian vegetation [23]. The study site is located within irrigated croplands occupying much of the palaeolagoon basin, while cereal fields, vineyards and other tree crops are also grown in the drier soils of the floodplain (Fig 1A). Freshwater marshes are characterized by bulrush (*Typha* spp.), sedges (*Scirpus* spp.), and the common reed (*Phragmites australis*). Surrounding the latter, patches of salt marshes dominated by marsh samphire (*Salicornia* spp.) and seepweed (*Suaeda* spp.) develop on saline, alkaline soils [23].

Coastal Mediterranean woodlands dominated by thermomediterranean plants such as holm oak (*Quercus ilex*), mastic tree (*Pistacia lentiscus*) and *Phillyrea angustifolia* develop in the calcareous mountain ranges bordering the flood plain to the west up to 800 m a.s.l. [25, 26] (Fig 1A). In the lower parts of these ranges and close to the agricultural zones, Aleppo pine (*Pinus halepensis*) woodlands and kermes oak scrublands (*Quercus coccifera*) develop on calcareous dry soils. In the lower mountain belt of the Pyrenean Albera Massif, woodlands dominated by cork oak (*Quercus suber*) expand over siliceous soils between 400 and 700 m a.s.l. At higher altitudes, stands of submediterranean woodlands with *Quercus pubescens* and *Fagus sylvatica* occur in wetter and cooler areas. Coastal scrub with rockrose (*Cistus monspeliensis*) and tree heath (*Erica arborea*) dominates in the easternmost part of the massif, extending to the Cap de Creus Peninsula (Fig 1A).

### Archaeo-historical context

The earliest evidence for stable occupation in the Alt Empordà dates back to the 5^th^ millennium BC. Neolithic open-air villages were occupied intermittently between 5000 and 1500 cal BC in the low-elevation ranges bordering the flood plain [27, 28]. Concomitant to this, a high concentration of Megalithic tombs are recorded in the Albera Massif, Serra de Rodes and Cap de Creus Peninsula [28]. Between 1300 and 600 cal BC Megalithic burials were replaced by large cremation urnfield cemeteries, while only one open-air site is recorded in the Albera Massif [29]. Commercial contact with Greeks and Phoenicians began after 700 cal BC [30], which soon resulted in the foundation of two Greek colonies in the Empordà coastline: *Emporion*, founded in 580 cal BC, and *Rhode*, founded at the beginning of the 4^th^ century BC [31] a few km east of the Castelló lagoon (Fig 1A). At the same time, the development of the Iron Age Iberian culture is attested, with large stable villages and hillforts documented in the basin between the 7^th^ and 3^rd^ centuries BC [31]. The Roman conquest during the 2^nd^-1^st^ centuries BC led to marked population expansion and intensification of activity in the Empordà plain. The city of *Emporiae* (Fig 1A) controlled and exploited the region through a network of villas [32]. A new period of economic progress began after the 5^th^ century AD under Visigothic rule, when the harbour city of Roses (Fig 1A) became the socio-economic centre of the region [33]. Moorish control of the territory during the 7^th^ and 8^th^ centuries AD resulted in the near-abandonment of settlements on the plain, as populations concentrated in the Serra de Rodes [33]. After the Carolingian conquest in 785 AD, the Empordà became a buffer zone between Christian- and Muslim-ruled territories. Christian colonization and the development of feudalism during the Medieval period led to renewed population growth and agrarian exploitation of the Empordà plain, with the appearance of fortified towns such as Castelló d’Empúries in the vicinity of the lagoon (Fig 1B) and of dispersed rural farms or “cortals” mainly oriented to the grazing exploitation of the alluvial plain [34]. A phase of marked political and economic splendour began after the 11^th^ century, when Castelló d’Empúries became the capital of the County of Empúries and developed as one of the most prosperous cities in north-eastern Catalonia [33]. This was mainly due to the expansion until the 16^th^ century of a commercial- and industrial-oriented economy dominated by salt production, flour and fulling milling, and textile manufacture [35].

## Materials and Methods

### Coring and sampling

Permit to conduct the drilling was issued by Josep Espigulé, director of the Empordà Wetlands Natural Park where coring was conducted. In 2009, coring was carried out in different sectors of the Castelló lagoon using a 50 cm x 5 cm ‘‘Russian” corer. Five cores were taken within the area covered by the palaeolagoon basin as defined by mid-18^th^ century maps [19] (Fig 1B). The exact location of the cores is presented in S1 Table. Cores were described in the laboratory according to sediment texture, grain size, and the presence/absence of macrofossils. The longest (636 cm-depth) core was chosen for palaeoenvironmental analysis: Estany d’en Mornau (42°16’ 53.6” N, 03°05’ 58.3” E), hereinafter EM (Fig 1B). Paleoecological analyses were performed in the 500 cm-depth muddy basal unit expanding below 160 cm-depth. One-cm thick subsamples were taken for multi-proxy analysis at intervals of 10 and 5 cm. Phases of marked environmental change were subsequently analysed at a higher resolution of 2 cm mainly between 340–355 and 415–475 cm-depth.

### Chronology

The chronology for EM was established using a variety of techniques. Historical maps and written sources were used to assign the uppermost calendar age. The core was dated by accelerator mass spectrometry (AMS) ^14^C measurements at Beta Analytic Inc. (Miami, USA). For the lower and upper part of the core, four dates were obtained on terrestrial plant remains and wood fragments. Dates were calibrated with CALIB 7.1 using the IntCal13 ^14^C calibration curve [36]. For the middle part of the core four radiocarbon dates were obtained from *Cerastoderma* sp. shells. Dates were calibrated and corrected for marine reservoir effect with the MARINE 13 calibration curve applying the nearest ΔR offset of 121±35 years, derived from modern pre-bomb seashell dates in Banyuls-sur-Mer, Southern France [36, 37]. The age-model was developed with CLAM v. 2.2 using a smoothing spline to the median age of each calibrated radiocarbon date [38]. In order to test the accuracy of the marine reservoir correction parameters, we compared the ^14^C dates of *Cerastoderma* sp. shells with dates derived from stratigraphically-associated ceramic imports in two undisturbed archaeological levels from the nearby site of *Emporiae* (Fig 1A). The archaeological levels were selected to avoid ^14^C plateaus. Dating of ceramic imports was performed by Tremoleda et al., [39] and P. Castanyer [personal communication] through typological analysis. Prior to ^14^C dating, shells were washed carefully with deionized water to remove any adhering organic acids and secondary carbonate. According to X-ray diffraction analyses, shell samples were composed of pristine aragonite, without secondary deposition or recrystallization. ^14^C AMS dates were calibrated and corrected for the marine reservoir effect as for shells from the EM core.

### Mineralogy and geochemistry

Oven-dried samples at 60°C were sieved at 250 microns and milled in an agate mortar for x-ray diffraction in order to determine the mineralogical composition: quartz, calcite, gypsum, pyrite, feldspar, and phyllosilicates (illite and chlorite), hereinafter reported as clays. 1 g of dried sediment was then used to calculate organic matter content by loss on ignition at 550°C in a muffle furnace [40, 41]. Subsequently, 0.1 g of these samples were processed for total acid dissolution in 25 ml PTFE beakers following Luo and Ku [42]. Geochemical analysis of elemental composition was performed at the U-series dating Laboratory of the Institute of Earth Science-CSIC using an ICP-AES (Inductively Coupled Plasma spectrometer). Geochemical values were normalised using the Ti content, an element rarely involved in biological and early diagenetic processes [43]. This contrast to Al, a more unstable element due to the occurrence of podzolisation processes in the study area [44]. Principal components analysis (PCA) of the geochemical data matrix was carried out using XLSTAT software v. 2015.5.01 [45].

### Ostracods

Ostracods and other biotic remains were studied on subsamples of ~10 g, processed according to methods described by Griffiths and Holmes [46]. Ostracod valves were counted under a stereomicroscope until a minimum number of 300 identifiable ostracod remains belonging to one species were counted, or the entire sample had been picked. Species were identified following specialized bibliography [47, 48, 49]. Densities (per gram dry weight) of all remains were transformed using log_10_(x+1) to avoid skewed distributions. In addition to ostracods, we also took into account the presence of charophyte gyrogonites, Gastropoda, cladoceran ephippia, Chironomidae and other insect remains, oribatid mites and Polychaeta (*Nereis* sp.). Four main groups of ostracods are distinguished in Table 1 using the known ecological tolerances of species.

**Table 1:** Ostracod groups and ecological interpretation as reported in specialized literature.

Ecological interpretation	Taxa	References
Euryhaline	*Cyprideis torosa*	[47, 50, 51]
Brackish	*Loxoconcha elliptica*, *Leptocythere lacertosa*, *Xestoleberis nitida*, *Cytherois stephanidesi*, *C*. *fischeri*	[47, 50, 51]
Marine	*Aurila arborescens*, *Xestoleberis communis*, *Loxoconcha rhomboidea*, *Leptocythere pellucida*, *Callistocythere littoralis*, *Semicytherura* cf. *striata*, *S*. cf. *robertsi*, *S*. cf. *acuticostata*, *Neocyhterideis* sp., cf. *Quadracythere* sp.	[47, 51]
Temporary fresh (and brackish)	*Sarcypridopsis aculeata*, *Heterocypris salina*	[48, 50, 52, 53]
Cold freshwater	*Fabaeformiscandona caudata*, *Physiocypria kraepelini*, *Metacypris cordata*, *Candona candida*, *Cyclocypris ovum*	[48, 49, 54]
Eurytherm and Mediterranean freshwater	*Candona* cf. *neglecta*, *Cypria ophtalmica*, *Mixtacandona* sp., *Pseudocandona* sp., *Limnocythere inopinata*, *Darwinula stevensoni*, *Cypridopsis vidua*, *Paralimnocythere psammophila*, *Herpetocypris* sp., *Vestalenula* cf. *danielopoli* group, *Ilyocypris gibba*	[48, 49, 50]
Other brackish-marine remains	*Nereis* sp., Gastropoda	[55]
Other freshwater remains	Charophyta, Cladocera remains, Chironomidae, Acari Oribatida	[56, 57]

### Diatoms

For diatom analysis, ~0.2 g of wet sediment was analyzed. Samples were prepared according to Battarbee [58]. Microscope slides were mounted using Naphrax^™^, and diatoms were counted at 1000x magnification. Approximately 500 valves per slide were counted in well-preserved phases and the presence or absence of dissolved fragments was noted elsewhere. Diatoms were identified using standard texts [59] and updated to the more recent nomenclature [60]. Diatom habitat classification follows Vos and Wolf [61], supplemented by the Spanish inland lake diatom-conductivity transfer function [62]. Diatom ecological groups are described in Table 2.

**Table 2:** Diatom groups and ecological interpretation as reported in specialized literature.

Ecological interpretation	Taxa	References
Marine plankton	*Chaetoceros* spp., *Actinocyclus* spp., *Paralia sulcata*, *Triceratium* sp.	[61, 62, 63]
Marine-brackish benthos	*Grammatophora macilenta*, *G*. *arcuata*, *Dimeregramma minuta*, *Toxarium undulatum*, *Nitzschia granulata*, *N*. *navicularis*, *N*. *lanceolatum*, *N*. *compressa* var. *vexans*, *Rhopalodia acuminatum*, *Opephora pacifica*, *Cocconeis scutellum*, *Amphora* sp. nov. cf. *marina*, *Diploneis bombus*	[61, 62, 63]
Brackish-fresh benthos	*Navicula rhyncocephala*, *Amphora pediculus*, *Cocconeis placentula*, *Staurosirella pinnata*, *Staurosira binodis*, *Fragilaria bronkei*, *Tabularia fasciculatam Fragilaria vaucheriae*, *Nitzschia angustata*	[61, 62, 63]
Fresh plankton	*Cyclostephanos invisitatus*	[61, 62, 63]
Fresh benthos	*Achnanthidium minutissimum*, *Planothidium lanceolatum*, *Synedra ulna*, *Fragilaria capucina*, *Hippodonta capitata*, *Navicula oppugnata*, *N*. *menisculus*, *N*. *antonii*, *Navicula* sp., *N*. *cryptotenella*, *N*. *capitoradiata*, *N*. *radiosa*, *Sellaphora bacillum*, *Amphora copulata*, *Cymbella affinis*, *Encyonema silesiacum*, *E*. *caespitosum*, *Encyonopsis microcephala*, *Gomphonema truncatum*, *Gyrosigma spenceri*, *Nitzschia amphibia*, *Diatoma elongatum*, *Epithemia sorex*, *E*. *adnata*, *Reimeria sinuate*, *Encyonema minutum*, *Cymbella helvetica*	[61, 62, 63]
Brackish-fresh aerophilous	*Luticola mutica*, *Hantzschia amphioxys*	[61, 62, 63]

### Pollen, non-pollen palynomorphs (NPPs), and charcoal

Pollen and NPP analysis were performed following standard procedures [64]. Microfossils were identified on a light microscope at 400x and 1000x magnification, and macrocharcoal particles >200 μm were tallied using a binocular microscope at 80x magnification. Pollen and NPP identification followed published illustrations and morphological keys [65, 66, 67], and the University of Barcelona′s pollen reference collection. At least 400 terrestrial pollen grains were counted per sample, together with the sum of NPP types identified during pollen counting. Pollen and NPP values are expressed as percentages of the total terrestrial pollen sum, which excludes Cyperaceae, fern spores and aquatic plants. Pollen and NPP taxa were assigned to ecological groups in accordance with local vegetation descriptions and specialized bibliography (Table 3).

**Table 3:** Pollen and NPP groups and ecological interpretation as reported in specialized literature.

Ecological interpretation	Taxa	References
Freshwater hydrophytes	*Potamogeton*, *Myriophyllum*, *Nymphaea*	[23]
Freshwater helophytes	Cyperaceae, *Typha-Sparganium*-type	[23]
Halophytes	Chenopodiaceae	[23]
Riparian woodland	*Alnus*, *Salix*, *Ulmus*, *Corylus*	[23]
Apophytes (ruderal & nitrophilous)	*Plantago* spp., *Rumex* spp., *Urtica dioica*-type, Cichorioideae, Asteroideae, *Achillea*-type, *Thalictrum*, *Galium*-type, *Lotus*-type, *Trifolium*-type, Brassicaceae, *Polygonum aviculare*, *Papaver*	[23, 68, 69]
Coprophilous fungi	Obligate: *Sporormiella*-type/ Non-obligate: *Sordaria*-type, *Cercophora* sp., *Podospora*-type, *Coniochaeta* cf. *lignaria*, *Apiosordaria verruculosa*, *Delitschia*, *Trichodelitschia*, *Chaetomium*	[70,71]
Freshwater algae	*Botryococcus*, *Spirogyra sp*., *Zygnema*-type, *Gloeotrichia*-type	[70]
Freshwater fauna	*Mycodalyellia armigera*, *Assulina muscorum*, *Arcella* sp.	[72,73]
Marine-brackish fauna	Microforaminiferal test linings, *Spiniferites* spp., *Lingulodinium* spp.	[74,75]

Stratigraphic diagrams of the various proxies were prepared using C2 software v. 1.7.2 [76]. Zonation was established using constrained incremental sum-of-squares cluster analysis using CONISS [77].

## Results

### Age-depth model

**Fig 2:**
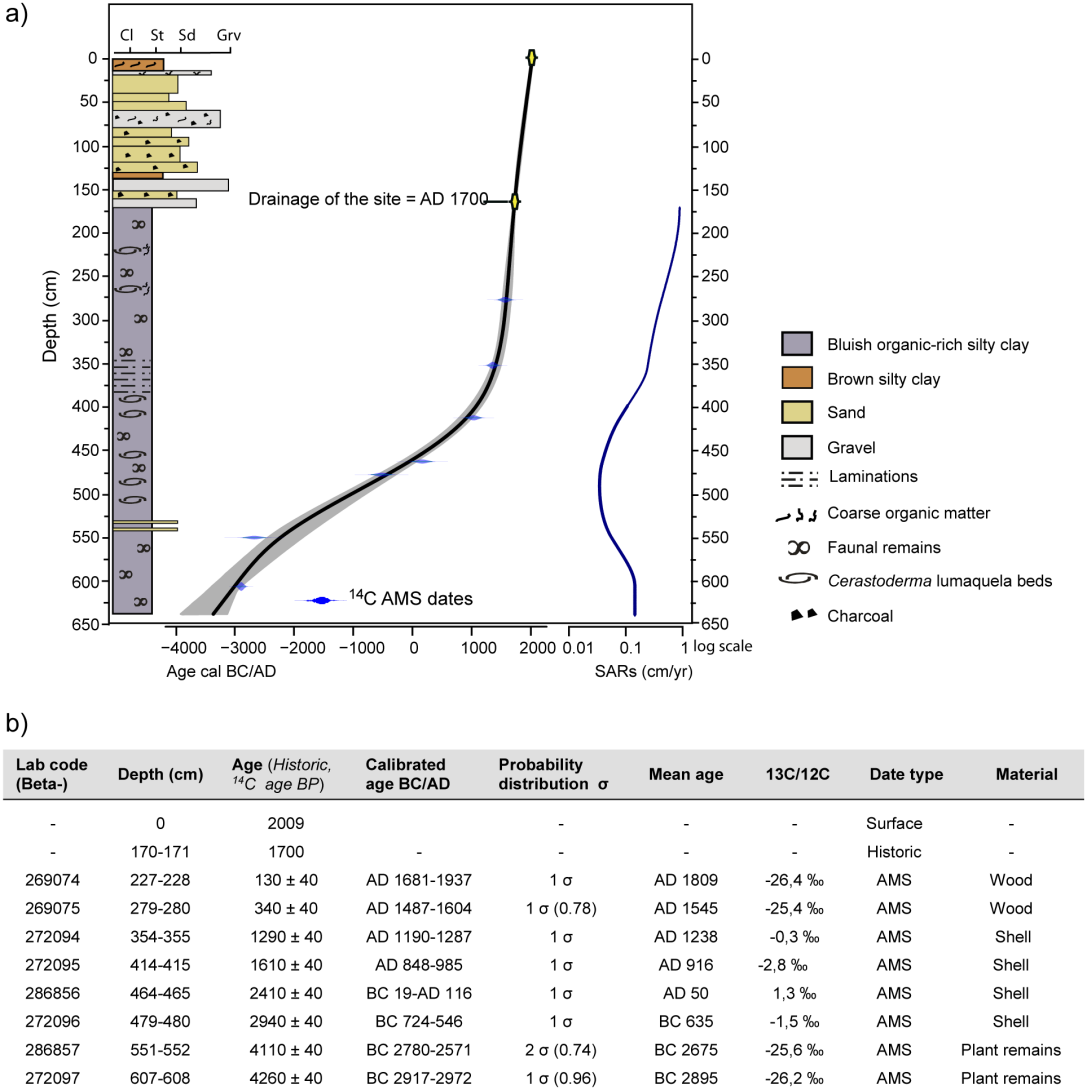
Age-depth model and dating results. A) Stratigraphy, age-depth model and sediment accumulation rates (SARs) for the EM core. (B) Results of direct radiocarbon and indirect historic dating

Fig 2 summarizes relevant historical data and results of ^14^C dating, including the age-depth model for EM, defined by seven radiocarbon dates and the uppermost historic date. These dates occur in stratigraphic order supporting continuous sedimentation from ~3150 cal BC to the 18^th^ century. The uppermost fluvial sands and gravels in the EM core are related to the expansion of the Mugueta lobe in the Castelló lagoon. According to ancient maps [78], the study area was already infilled by the second half of the 18^th^ century AD (Fig 1B). However, written sources from the late 17^th^ century AD document the existence of new farmed alluvial deposits or “aigualleixos” in the ancient lagoon basin, ~1 km to the west of EM [18], supporting an earlier date for this fluvial infilling process. This evidence suggests that the EM site was infilled between the late 17^th^ century and the 2^nd^ half of the 18^th^ century, and thus we have assigned an indirect date of ~1700 AD at 170 cm-depth. The ^14^C date at 228 m-depth of ~1800 AD is inconsistent with the documented infilling process. For this reason, we have not included this date in the age-model.

The results of ^14^C dating of *Cerastoderma* sp. shells and pottery from archaeological levels from the *Emporiae* site are compared in Table 4. After correction and calibration, shell ^14^C dates were only between 30 and 125 years younger than pottery chronologies. This age difference falls within the standard deviation of the calibrated ^14^C dates on shells used to build the age-depth model of the EM core (Fig 2B). This supports the accuracy of the marine reservoir correction made on shell dates using the ΔR offset for this region, and the reliability of our age-depth model from 480 to 355 cm-depth.

**Table 4:** ^14^C dates of paired *Cerastoderma* sp. shells and ceramic imports in archaeological levels.

Shell (*Cerastoderma* sp.)						Pottery	
Stratum	^14^C lab code (Beta-)	Age ^*14*^*C yr BP*	Calibrated age BC/AD 1 σ	Calibrated age BC/AD 2 σ	^13^C/^12^C	Age	Reference
Sample 1 99-Cr-Cb-1032	402313	2440 ± 30	BC 35—AD 75 *Mean*: *AD 20*	BC 95—AD 120 *Mean*: *AD 13*	-1,3 ‰	10–20 AD *Mean AD 15*	[39]
Sample 2 94-SM-5121	402314	2200 ± 30	AD 245–355 *Mean*: *AD 300*	AD 175–410 *Mean*: *AD 285*	-7,3 ‰	AD 400–450 *Mean*: *AD 425*	Castanyer 2015 (personal communication)

Based on the age-depth relationships, sediment accumulation rate (SAR) has not been constant over time (Fig 2A). SAR is low from the core base to 595 cm-depth (averaging ~0.15 cm/yr), and reaches a minimum of ~0.03 cm/yr between 595 cm and 490 cm-depth. An increase to ~0.24 cm/yr occurs between 490–350 cm-depth, followed by a marked increase to ~0.9 cm/yr in the upper sequence.

### Mineralogy and geochemistry

**Fig 3:**
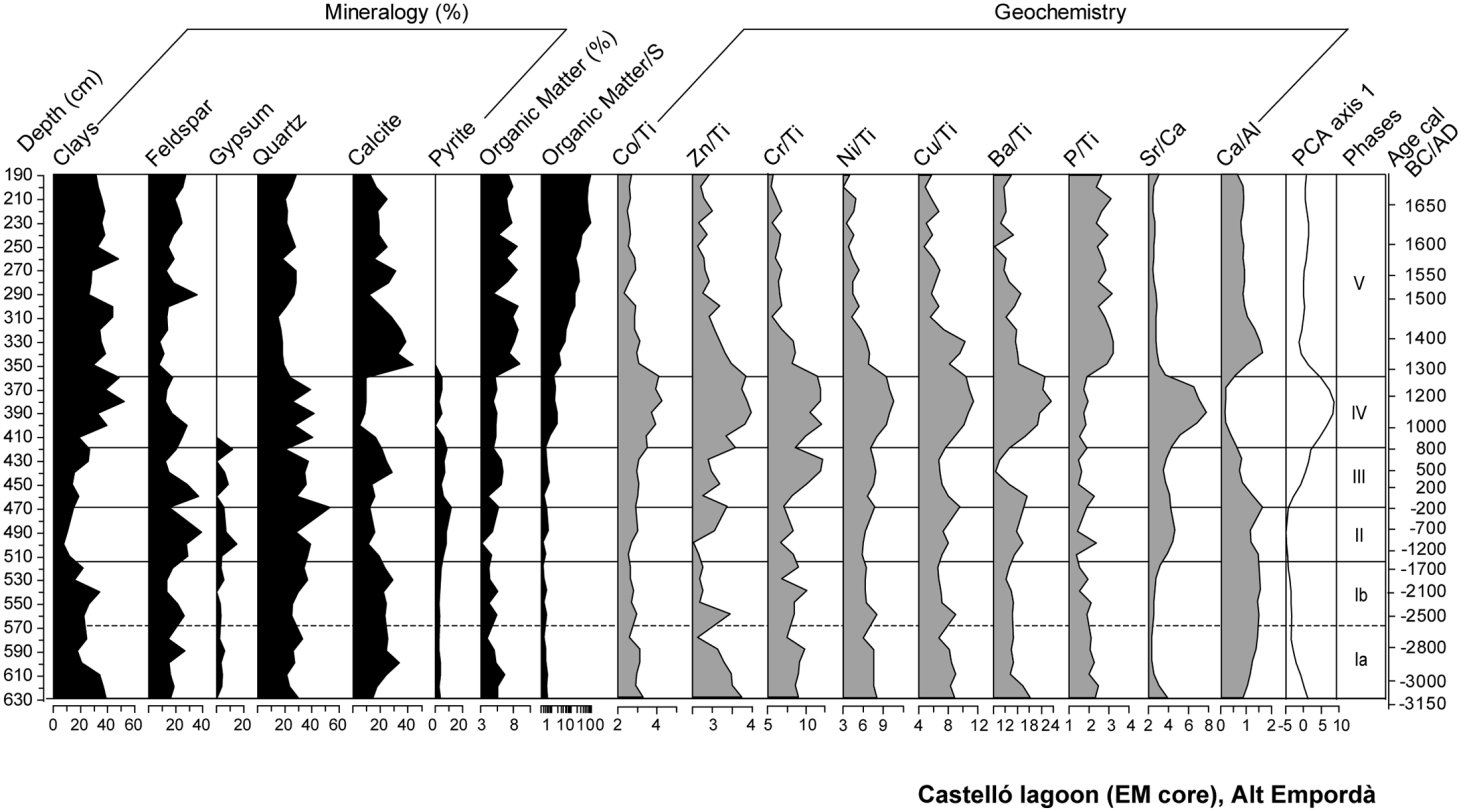
Mineralogy results, organic matter content and selected geochemical proxy ratios for EM. The last column corresponds to sample distribution according to the first eigenvectors of PCA.

**Fig 4:**
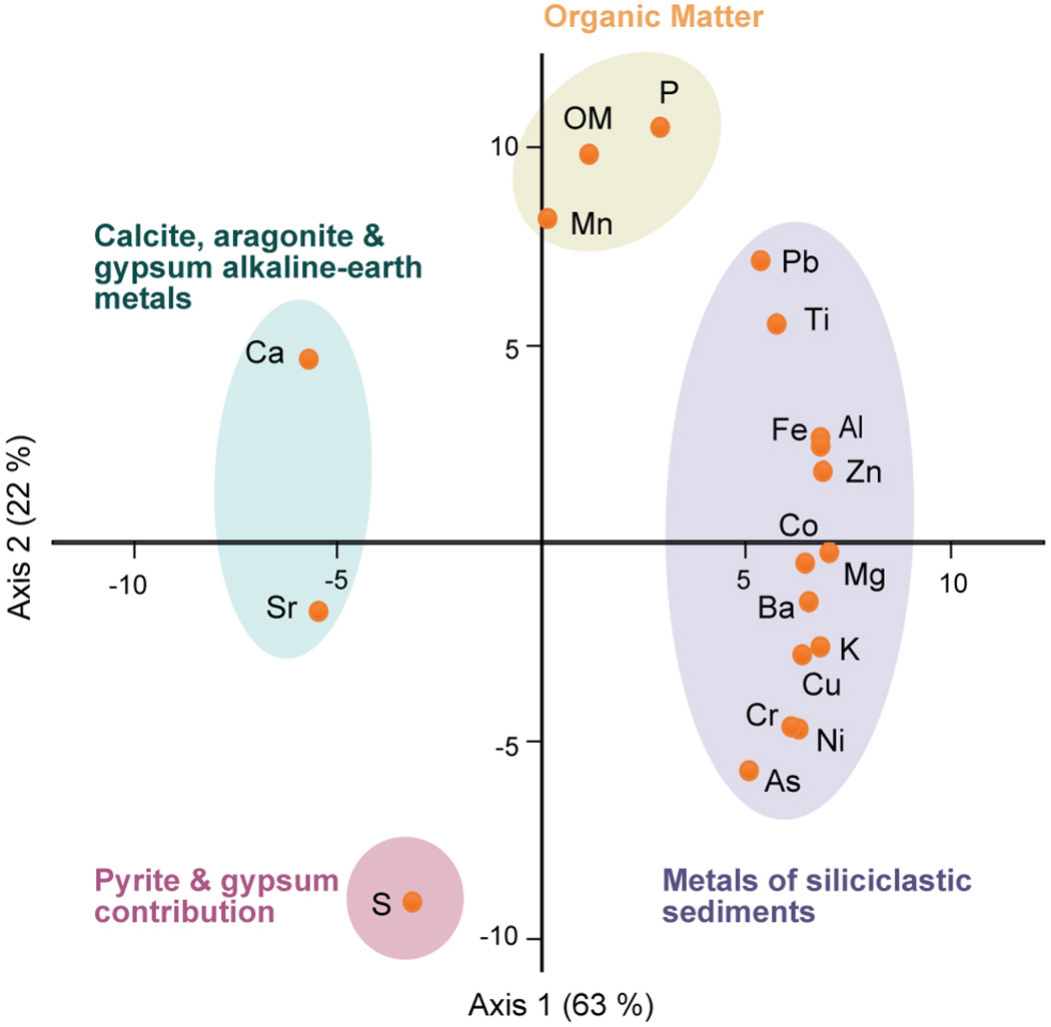
Distribution of elements and Organic Matter content according to PCA analysis.

The lithostratigraphy of the 636 cm-depth EM core consists of a basal unit of 500 cm of muds with interbedded sands containing brackish macrofossils such as *Cerastoderma* sp., overlain by 170 cm of fluvial gravels, sands and muds with terrestrial gastropods (Fig 2A). Fig 3 shows the mineralogical and normalized elemental composition of EM and Fig 4 the PCA results. The elemental concentrations of studied bulk elements is presented in S2 Fig. The mineralogy of the cores as determined by XRD analysis shows that the dominant carbonate in the sediments is calcite. The second authigenic dominant mineral is gypsum, showing the highest abundance during the lower three meters of the core. Both minerals in the sediments are most likely formed as primary precipitates from the water column. Pyrite occurs in the anoxic lower part of the record. The clastic fraction is composed almost entirely of chlorite, illite, biotite, quartz and feldspars. The distribution of the variables in relation to axis 1 (63% of the variance) indicates an opposition between silicate-related elements (Ti, Al, K, Pb, Cu, Fe, Mg, Zn, Ba, Co, Ni, Cr, As) and alkaline-earth metals related to calcite, aragonite and gypsum (Ca, Sr). The second axis (22% of the variance) opposes organic matter (OM), P and Mn to S, which mainly corresponds to gypsum and pyrite content.

### Palaeoecology results

Biotic remains are generally well preserved apart from diatoms, these being well preserved only in the phases 620–575 cm and 355–280 cm-depth. However, dissolved, uncountable fragments of marine-brackish diatoms intermittently occur between 425 and 575 cm-depth.

**Fig 5:**
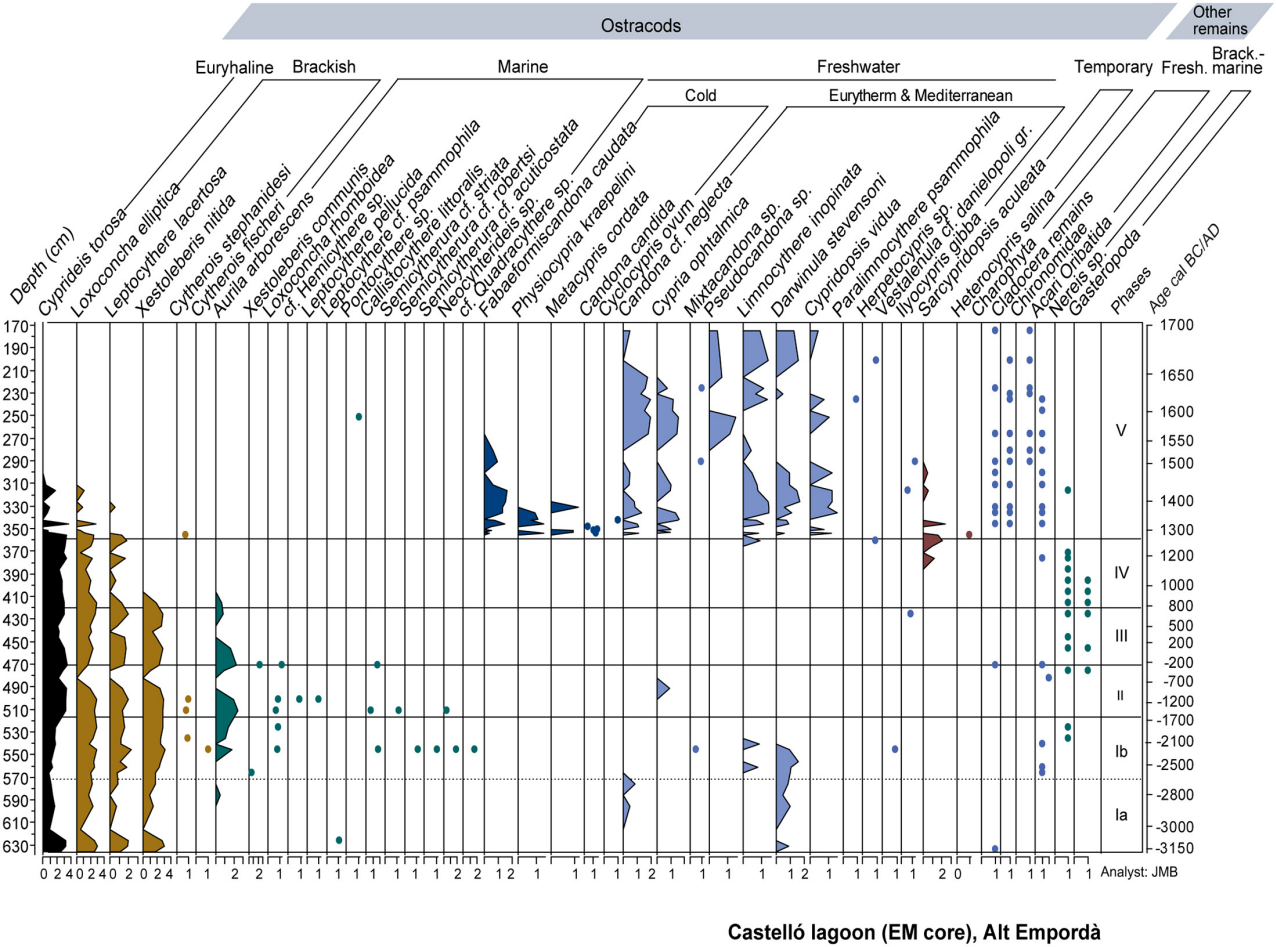
EM ostracod and other biotic remains diagram showing species concentration values transformed using log_10_ (x+1).

**Fig 6:**
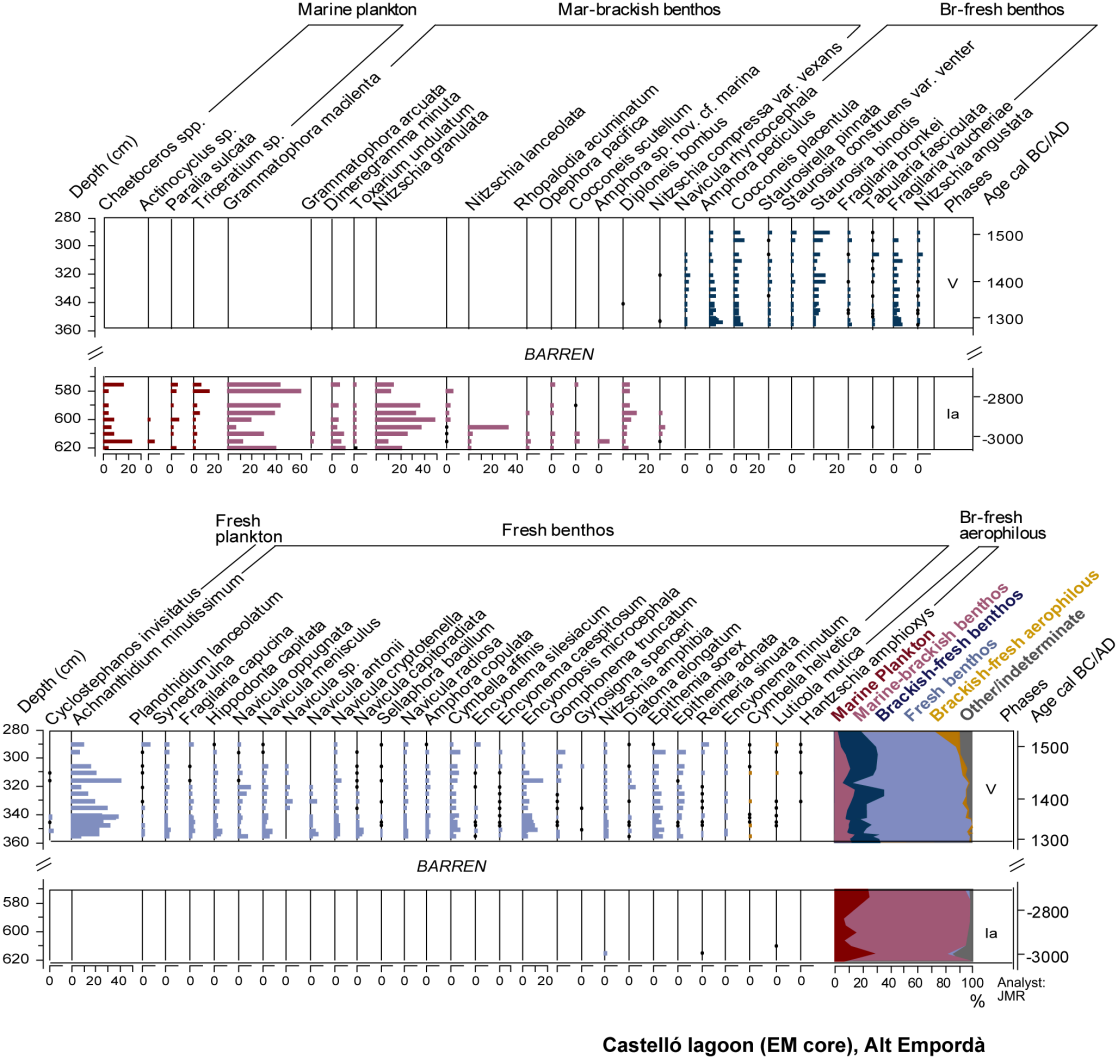
EM summary percentage diatom diagram.

**Fig 7:**
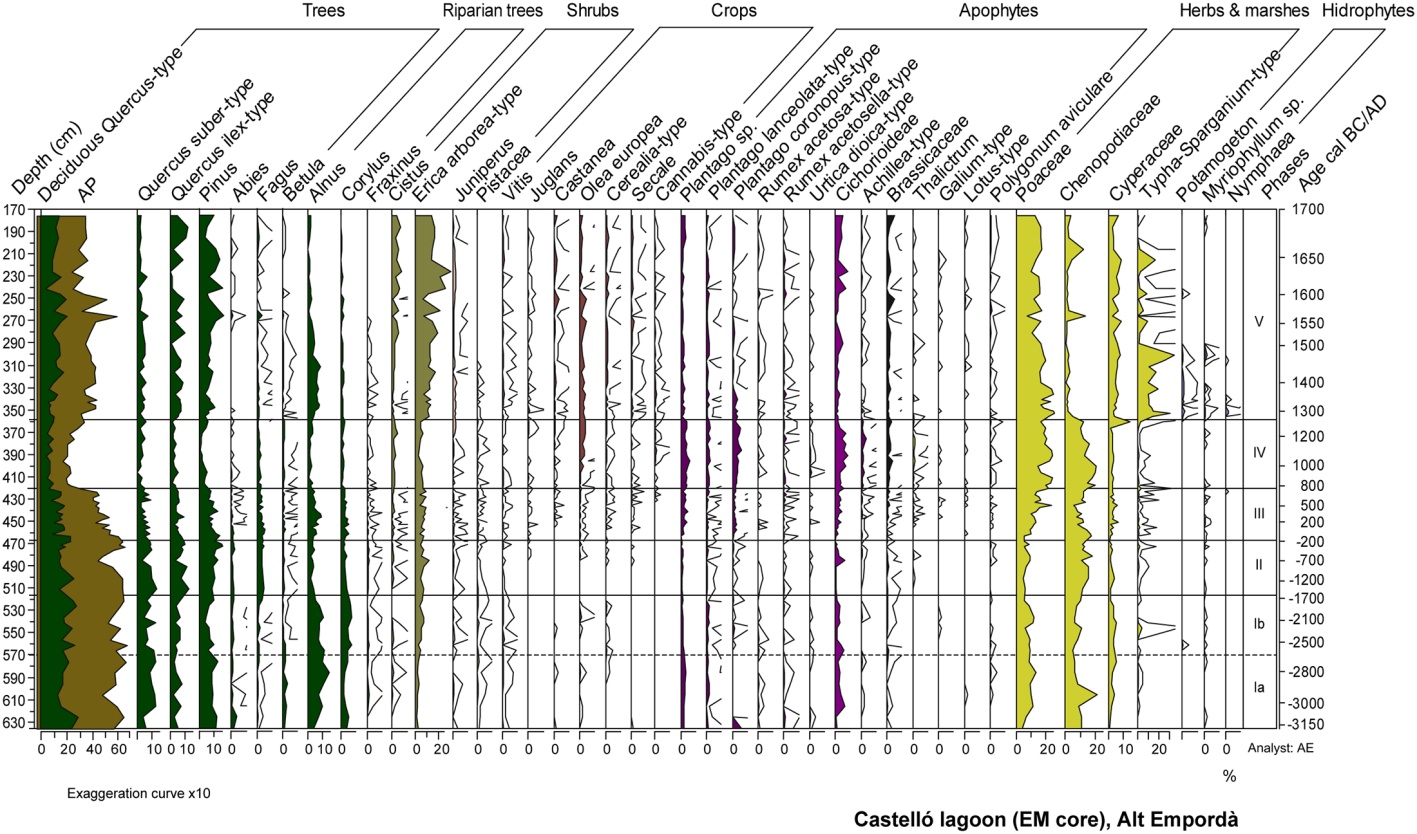
Selected pollen percentages for EM.

**Fig 8:**
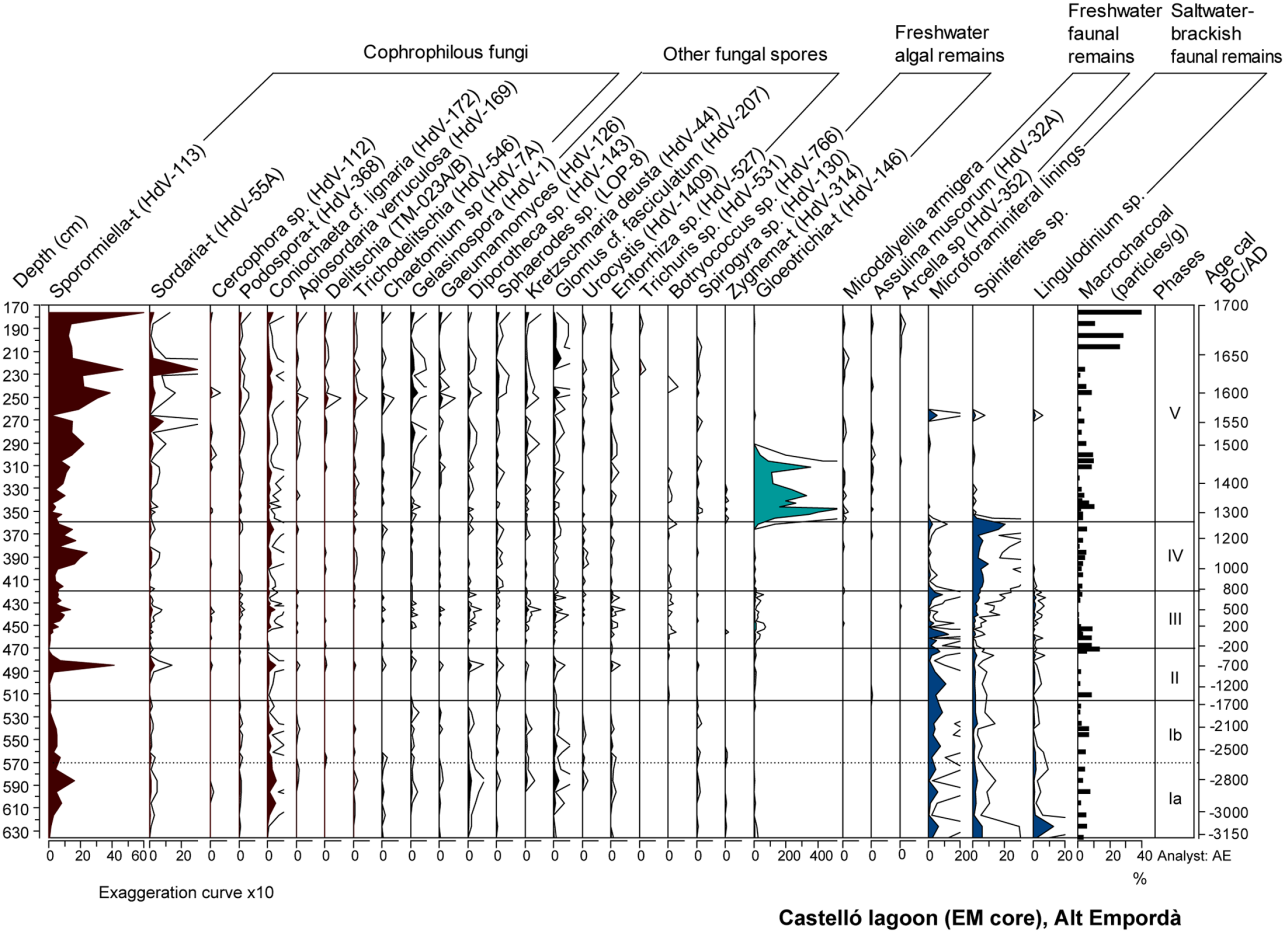
NPPs percentages and raw macrocharcoal concentration values for EM.

**Fig 9:**
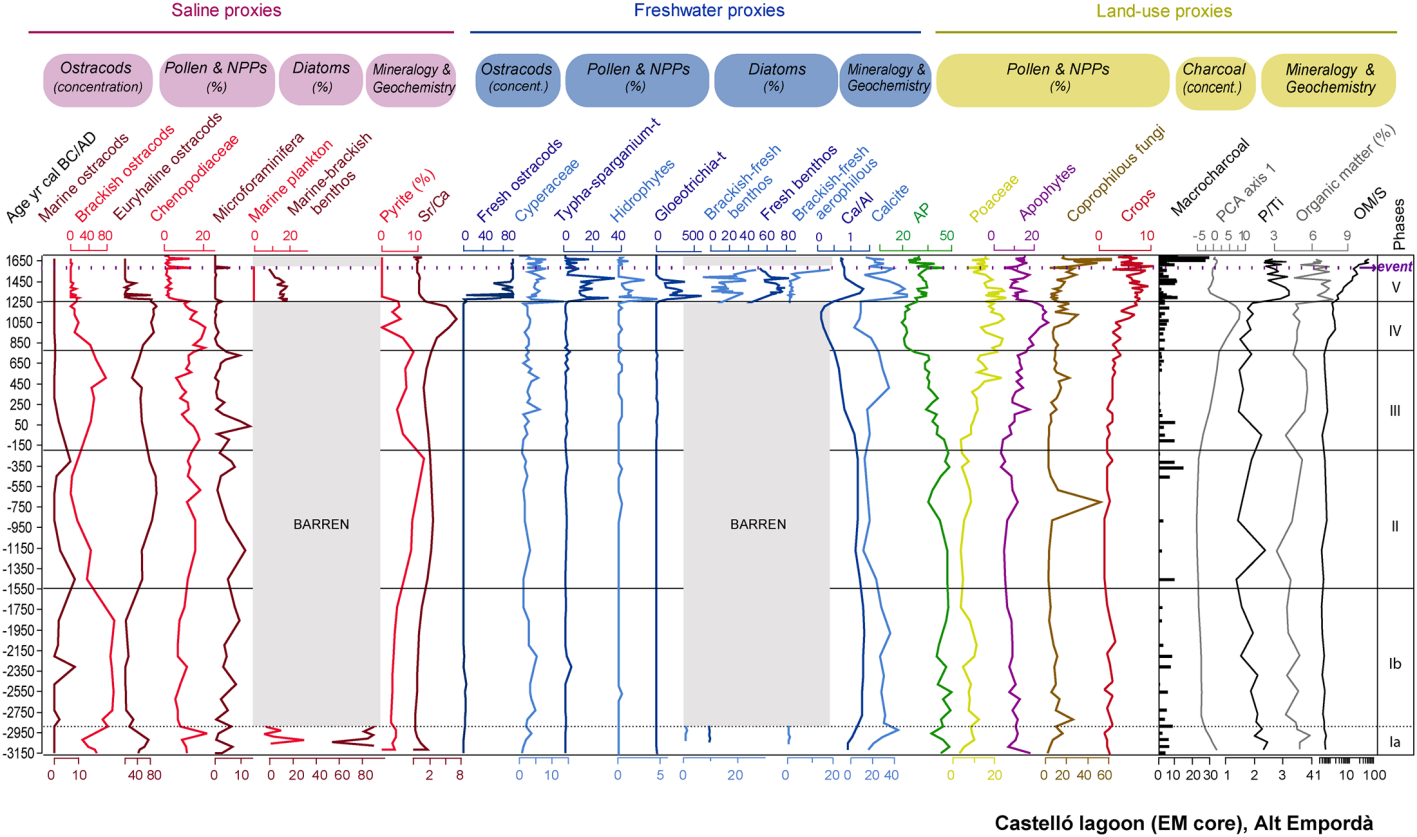
Composite diagram including key selected variables, showing the inferred sensitivity to lake salinity and land-use changes. Ostracods curves are expressed in concentrations transformed using log_10_(x+1).

Ostracod and diatom results are displayed in Figs 5 and 6. Pollen and NPPs results are shown in Figs 7 and 8. The correlation of zones established for the various studied proxies on the basis of CONISS cluster analyses is shown in S1 Fig. The consistency between zone boundaries for the EM record is used to establish a total of five common environmental phases. Fig 9 displays a composite diagram with key selected variables of lake salinity and land-use changes

#### Phase I a/b (635–515 cm, ~3150–~1150 cal BC)

The lithological composition of this phase is characterized by fine grained sediments with interbedded sandy layers and abundant faunal remains such as foraminifera and molluscs (Fig 2A). Amongst the latter, *Cerastoderma* sp. shells, some of them recovered in their living position, occasionally form 5-cm thick shell beds. Organic matter content ranges between 3–6%, and the presence of gypsum and pyrite is observed (Fig 3). Changes in the ostracod and diatom assemblages allow differentiation of two subphases. Subphase 1a is defined by the presence of euryhaline ostracods and marine and brackish diatoms (Figs 5 and 6). During subphase 1b, brackish ostracods expand over euryhaline species, with the occasional occurrence of marine ostracods. Freshwater ostracods occur at low abundance in both subzones, in particular *Darwinula stevensoni*, which is known to dwell together with euryhaline brackish species *C*. *torosa*, *L*. *elliptica* and *X*. *nitida* in low salinity, permanent water bodies of Mediterranean coastal areas [50]. Towards the top of subphase 1b there is a clear increase of marine ostracod remains. Amongst pollen and NPP taxa, halophytes (Chenopodiaceae) and faunal remains such as microforaminiferal linings and dinoflagellate cysts (*Spiniferites* spp., *Lingulodinium* spp.) are well represented in subphase 1a, while the rise of freshwater helophytes (Cyperaceae, *Typha-Sparganium*-type) is observed during subphase 1b. The sporadic occurrence of freshwater hydrophytes and algal spores such as *Myriophyllum* sp., S*pirogyra* sp. and *Zygnema*-type occur in both subzones (Figs 7 and 8).

The pollen spectrum is dominated by arboreal pollen (AP), at up to 69% abundance, mainly composed of deciduous *Quercus-*type, *Quercus suber*-type, *Alnus* and *Pinus*. *Quercus ilex*-type and *Corylus* and to a lesser extend *Betula* and *Abies* are also common. Recurrent AP declines up to down to 50–56% occur during the phase, comprising the significant retreat of deciduous *Quercus*-type down to 13% during subphase 1a, and of a more varied range of tree taxa including also *Quercus suber*-type, *Alnus*, and *Fagus* during subphase 1b. These retreats are coeval with slight rises in herbs, *Erica arborea*-type and apophytes–i.e. Cichorioideae, *Plantago lanceolata*, *Urtica dioica*, *Rumex acetosa*-type-, and with the regular presence of Cerealia-type at low values <1% during subphase 1b. The NPP record shows the increase in dung-related fungi throughout the phase, particularly of *Sporormiella*-type up to 16% at 585 cm-depth. Macrocharcoal concentration values up to 9 particles/g are recorded along the phase (Fig 8).

#### Phase II (515–467 cm, ~1550–~150 cal BC)

Sediments are formed by silts with the occurrence of successive *Cerastoderma* sp. shell beds. According to X-ray diffraction analysis, sediments are characterized by high contents of quartz and feldspar with a minor contribution of clays and calcite (Fig 4). This phase shows maximum values of pyrite and gypsum up to 11% and 15%, respectively (Fig 3). The geochemical change in the sediment composition is well represented in the PCA-F1 by minimum Axis 1 scores (Fig 4). Brackish and marine ostracods show their highest abundances and diversity in this zone, and freshwater ostracods are rarely found (Fig 5). Diatom frustules are not well preserved in this phase, but rare fragments of dissolved taxa such as *Nitzschia granulata* suggest the flora was similar to Zone 1. Amongst herbaceous pollen taxa, the expansion of Chenopodiaceae up to 18% and the retreat of Poaceae are the main feature of the phase (Fig 7). A change in the woodland composition is also observed, with either the retreat or disappearance of riparian tree taxa and the rise of *Quercus ilex*-type, *Quercus suber*-type, *Pinus* and *Fagus*. A marked retreat of AP down to 47% is recorded between 500 and 485 cm-depth. This represents an initial retreat of deciduous *Quercus*-type from 26 to 16%, followed by the fall of *Q*. *ilex*- and *Q*. *suber*-types down to 3% between 480 and 485 cm-depth. This retreat in evergreen oaks is coeval with a rise of Poaceae, *Erica arborea*-type and apophytes (Cichorioideae, *Rumex* sp.). Cerealia-type and *Olea*, absent during the first half of the phase, reappear from 485 cm, together with the first presence of *Secale* and *Juglans*. All these crop pollen types show low values <1%. A peak of *Sporormiella* up to 41% and of other coprophilous fungi is also observed (Fig 8).

#### Phase III (467–421 cm, ~150 BC—~750 cal AD)

Mineralogical and geochemistry results indicate the rise of calcite and Al, the fall of the Sr/Ca ratio and the continuous presence of gypsum (Fig 3). The geochemical change in the sediment composition is well represented in the PCA with a progressive rise in the F1 scores (Fig 4). This phase shows the expansion of brackish ostracods and the progressive retreat of marine taxa (Fig 5). The pollen record shows increasing trends of Cyperaceae and the retreat of Chenopodiaceae down to 6% (Fig 7). *Typha*, *Gloeotrichia*-type and *Botryococcus* are more abundant than previously. A progressive retreat in AP down to 48% is also observed. This affects mostly *Quercus* taxa and *Pinus*, all showing saw tooth-like curves. Conversely, *Alnus* and to a lesser extent *Corylus* recover. A progressive increase of Poaceae from 9% to 27%, the increase of apophytes and a rise of dung-related fungi also occur. Amongst crop taxa, the regular recording of *Castanea* and *Cannabis*-type start since 453 cm- and 430 cm-depth, respectively. Finally, macrocharcoal particles rise at the base of the phase up to 9 particles/g (Fig 8).

#### Phase IV (421–362 cm, ~750–~1250 cal AD)

The lithology of the phase is formed by abundant shell beds in a muddy matrix. Fine laminated beds occur at 380 cm-depth (Fig 2A). Sediment composition shows a high contribution of clays and quartz, the lowest values of calcite, and the disappearance of gypsum. Also, the Sr/Ca ratio shows maximum values, the Ca/Al ratio falls down to minimum values, P rises and a peak of the organic matter/S ratio is observed (Fig 3). This geochemical change is well attested by maximum scores in the PCA F1 (Fig 4). Maximum values of heavy metals (Ni, Cu, Fe, Co, Zn and Ba) are recorded. The ostracod assemblage is characterized by the disappearance of marine species and dominance of a few brackish and euryhaline taxa, together with ostracods typical of temporary brackish-to-freshwater environments (*Sarscypridopsis aculeata*, *Heterocypris salina*) at the top of this phase (Fig 5). The pollen record shows both the expansion of Chenopodiaceae up to 20% and lower values of Cyperaceae (Fig 7), while *Lingulodinium* spp. is replaced by *Spiniferites* spp. within the NPP assemblage (Fig 8). AP retreats, reaching minimum values of the record of 25%. This represents the fall of most tree taxa including *Quercus* spp., *Pinus*, *Abies* and riparian trees. The retreat of *Erica arborea*-type also occurs. The pollen assemblage is now dominated by herbaceous taxa, with Poaceae increasing to 25% and apophytes reaching maximum values of the record up to 26% (Fig 9). Amongst crop taxa, *Olea* and *Cannabis*-type also rise up to 4 and 1%, respectively. Amongst NPPs, *Sporormiella*-type rises to 24%. Charcoal particles are present throughout, peaking at 6 particles/g.

#### Phase V (362–175 cm, ~1250 cal AD—~1700 AD)

The lithology comprises massive silty clay sediments containing occasional roots and molluscs, with high values of calcite content (up to 40%) in the first part of this phase. Pyrite disappears and the Sr/Ca ratio is low, corresponding with low PCA F1 scores. A major rise of organic matter content and P occurs, together with a rising trend in the organic matter/S ratio (Fig 3). A major change is observed in the ostracod assemblage, with the appearance and dominance of freshwater species and the disappearance of brackish and euryhaline taxa (Fig 5). The presence of cold freshwater species, typical of continental Europe and/or high altitudes [48, 49, 54] is documented in the lower half of the phase. A similar change to freshwater communities is observed in the diatoms, with diverse, well-preserved assemblages dominated by the freshwater benthic diatom *Achnanthidium minutissimum*, and a range of others such as *Epithemia sorex*, *Cymbella microcephala* and *Reimeria sinuate* (Fig 6). Marine taxa are absent and ‘brackish-fresh’ benthos which tolerate moderate salinity are at a low abundance. The presence of the highly planktonic diatom *Cyclostephanos invisitatus* is combined with a mildly euthrophic benthic flora (Fig 6), while the presence of charophyte gyrogonites typical of clear waters is also observed [79] (Fig 5). Diatoms are poorly preserved above 290 cm-depth, after a marked increase in freshwater aerophilous taxa such as *Luticola mutica* in the uppermost sample. A significant change occurs within wetland pollen taxa, with the replacement of Chenopodiaceae by freshwater helophytes and hydrophytes (Fig 7). Amongst the latter, the presence of *Potamogeton* sp., *Myriophyllum* sp. and *Nymphaea* sp. indicates a permanent water column of about 1 to 3 m deep [80]. This is coeval with changes in the NPP assemblage, showing the sudden rise of *Gloeotrichia*-type, the presence of other freshwater algal spores (*Spirogyra*, *Zygnema*-type) and faunal remains (*Micodaelyellia armigera*, *Assulina muscorum)* and the disappearance of dinoflagellate cysts and microforaminiferal linings (Fig 8).

The pollen record is characterized by the recovery of AP up to 63%, driven mainly by deciduous *Quercus*-type, *Pinus*, *Quercus ilex*-type and *Alnus* (Fig 7). Shrub taxa also expand, mainly *Erica arborea*-type (up to 30%) and *Cistus* (up to 7%). Apophytes experience significant retreats below 10% along the zone (Fig 9), while some crops rise: *Castanea* (up to 3%), Cerealia-type and *Secale* (up to 2%), and *Vitis* (up to 1%). Coprophilous fungi rise, with *Sporormiella*-type reaching maximum values of the record up to 60% and a peak of *Sordaria*-type. Coeval to this, parasite eggs (*Trichuris* sp.) appear. Highest values of macrocharcoal up to 40 particles/g occur towards the uppermost part of the phase boundary (Fig 8).

A well-defined, short-lived event occurs at ~266 cm-depth (~1560 AD). The deposition of *Cerastoderma* shells (Fig 2A) coincides with a decrease in the OM/S ratio, and a peak of Chenopodiaceae, microforaminiferal test linings and dinoflagellate cysts (Fig 9).

## Discussion: Environmental History and Human Management of the Castelló Lagoon and Its Surrounding Landscape

### Phase I a/b [~3150–~1150 cal BC]. *A lagoon in a wooded coastal plain used by late Neolithic groups for agropastoral purposes*

Between 3150 and 1550 cal BC (Fig 9), the biotic proxy data indicate the varying influence of waters ranging from fresh to brackish, but dominated by marine and brackish taxa indicative of a lagoon with strong marine influence. Extensive diatom dissolution in the upper phase is also consistent with the influence of high salinity [63]. Muddy beds with *Cerastoderma* sp. shells interbedded with sandy layers suggest that the lagoon was occasionally affected by over-wash fans from a distal island barrier [8]. After 2900 cal BC (610 cm) the gradual decrease in SAR (Fig 2A) may indicate a progressive sea level rise not compensated by sediment deposition at the study site. The occurrence at low abundance of freshwater indicators throughout Phase I supports the existence of fluvial water inlets in the lagoon. This combined influence of fluvial and marine water inputs would favour the existence of mosaic-like vegetation in these wetlands, supported by palynological data suggesting patches of salt and fresh-to-brackish sedges and reeds growing in the depressed marginal areas of the lagoon.

The lagoon was located within a forested landscape dominated by submediterranean woodlands mainly formed by deciduous oaks (*Quercus*-type), while pine (*Pinus*), cork oaks (*Q*. *suber*-type), and to a lesser extent holm oaks (*Q*. *ilex*-type), developed in nearby ranges (Fig 7). An alder-dominated riparian woodland would also grow in the nearby moist floodplain soils less exposed to soil salinity and along fluvial corridors. Despite this overall wooded landscape, moderate but recurrent fire-related openings resulted from agropastoral activities in the lagoon catchment between 3150 and 2000 cal BC (Fig 9). The use of coastal wetlands for grazing purposes is supported by rises in apophytes and abundance of dung-related fungal spores (Fig 8). Palynological data also indicate low-intensity cereal agriculture after 2900 cal BC. The agropastoral exploitation of coastal wetlands and landscapes resulted from increased regional human settlement, with the synchronous occupation of three nearby open-air villages between 3400 and 2700 cal BC: Serra del Mas Bonet, Camí dels Banys de la Merc00E9 and Ca n’Isach [27, 28], and with the first occupation of the Riera Masarac site between 2865 and 1980 cal BC [28] (Fig 1A). At the Serra del Mas Bonet site, the abundance of domestic faunal remains, and the recording of stoneware carved steles representing bovid horned heads [27] further supports the economic and cultural importance of grazing for late-Neolithic coastal communities. The existence of recurrent agropastoral openings within an otherwise wooded landscape is also evidenced in nearby palynological coastal records during the Neolithic. This is the case of the Sobreestany lagoon (Baix Empordà), where clearances were coupled with cereal cultivation since ~4700 cal BC [81]. A similar context is evidenced further to the north in the Gulf of Lyon (Languedoc, France): in the Palavás lagoon complex after ~2550 cal BC [15], and in the Thau lagoon since ~5000 cal BC, being in this latter site fire-induced openings related to grazing and cereal cultivation [14].

### Phase II [~1550–~150 cal BC]. *Maximum marine inundation and short-lived local human impact*

Between 1550 and 150 BC there is evidence for maximum marine flooding as a consequence of sea level rise. This is supported by minimum SAR at 880 cal BC, and the full dominance of marine and euryhaline ostracods and microforaminiferal linings (Figs 2 and 9). Condensed sections with very low SARs are typical of maximum marine-flooding episodes [82]. In addition, the increased presence of pyrite indicates anoxic bottom waters probably related to increased water depth and stratification. These conditions would favour the expansion of salt marshes (increase of Chenopodiaceae pollen) and the retreat of alder (*Alnus*), which are highly sensitive to soil salinity, in the surrounding mud flats (Fig 7). Sea flooding also resulted in reduced agropastoral activity within the lagoon catchment. This is suggested by the retreat of apophytes and dung-related fungi and associated woodland recovery (Fig 9) at a time when archaeological settlements located in the floodplain, such as Serra del Mas Bonet and Camí dels Banys de la Mercé Fig 1A, were abandoned [27]. From 1500 BC until the late Iron Age, the only clear evidence for human occupation in this area is found further inland in nearby ranges [29].

However, a short period of increased human activity is recorded from the 8^th^ to the 6^th^ centuries BC (480–485 cm-depth) (Fig 9). The renewal of grazing activities is supported by a peak in coprophilous fungi, the rise of apophytes and herbs (Fig 9). Concomitant reduced salinity is indicated by a decrease in microforaminifera, brackish ostracods and Chenopodiaceae, and the presence of freshwater ostracods (*Cypria ophtalmica*), algae (*Zygnema*-type) and hydrophytes (*Myriophyllum* sp.) (Figs 5 and 9). This freshening may have favoured renewed local grazing exploitation of littoral wetlands. Further evidence for increased human activity comprises clearance of evergreen oaks growing in bordering ranges (decrease in *Quercus ilex*-type and *Q*. *suber*-type trees, and the rise of *Erica arborea*-type heath), and palynological evidence for resumed regional cultivation including new crops. The latter comprises low values of cereal (Cerealia-type) and olive (*Olea*) and the appearance of walnut (*Juglans*) and rye (*Secale*). Indeed, the spread of new cultivars is characteristic of Iberian Mediterranean areas during the early Iron Age. For instance, millet, oats and cultivated vines are first reported in Iberian sites from the Baix Empordà since the 7^th^ century BC [83], while olive cultivation is documented in the south of France from the late Iron Age [84]. According to our age model, this coincides with the onset of cultural and commercial contacts of local populations with Phoenician and Greek cultures, as inferred from the study of the Agullana burial site [30] (Fig 1A), and evidence for the development of large stable Iberian settlements in the Baix Empordà during the 7^th^ century BC. The latter was soon followed by the establishment of the Greek colony of *Emporion* in the late 6^th^ century BC [31] (Fig 1A). Increased settlement together with commercial and cultural development in the Empordà basin most likely fostered the increased but shortlived agropastoral exploitation of coastal areas. Nevertheless, within the error range of the age model, there is also a possibility that this phase relates instead to the subsequent local development of the Iron Age Iberian settlements of Pontós and Perelada during the 6^th^ and 5^th^ centuries BC [31] and to the founding of the *Rhode* Greek colony during the early 4^th^ century BC (Fig 1A). In any case, neither the development of the Iberian culture nor the Greek colonization of this territory seem to have triggered major and long-lasting transformations of coastal landscapes.

### Phase III [~150 BC—~750 cal AD]. *A restricted lagoon and fluctuating marine influence*: *moderate human activity within a wooded landscape*

The shift in lagoon geochemistry to more dilute waters (low Sr/Ca ratio, rise of calcite) and an increase in siliciclastic sediments (Al) provides strong evidence for reduced marine influence (Figs 3 and 9). The Sr/Ca ratio is commonly related to water salinity in marine and lake sediments [85, 86], as well as in lagoons [87]. This gradual process begins at about 150 BC and involves the replacement of marine ostracods by brackish communities, reaching their maximum expansion at ~500 AD. This is consistent with the formation of a continuous sand barrier, with reduced marine influence. Reduced marine influence would favour the replacement of salt- by freshwater marshes, reeds and wet meadows (Cyperaceae, Poaceae, *Thalictrum*, *Galium*) in the lagoon’s marginal areas, together with a recovery of riparian woodland, as indicated by palynological data (Figs 7 and 9). After 500 AD, the expansion of euryhaline and brackish ostracods over more marine-restricted species suggests that the lagoon experienced salinity fluctuations, consistent with the behavior of a more hydrologically-closed system sensitive to intra- and interannual fluctuations in freshwater input. The rise of the Sr/Ca ratio points towards increased salinity, again consistent with brine concentration, and favouring the progression of halophyte vegetation and the retreat of riparian communities.

During phase III there is evidence for increased human impact, as shown by the progressive decrease in AP, the rise of apophytes and the renewal of grazing activities. There is also evidence for a more regular and diverse presence of crop pollen, including the first consistent presence of chestnut (suggested by the more regular recording in the genus *Castanea*). Increased agropastoral exploitation resulted from the intense rural occupation of this territory following the Roman colonization after the 2^nd^ century BC, when the Empordà plain was under the direct control of the Roman city of *Emporiae* (Fig 1A). Increased settlement and strong organization of the plain is attested by the recording of Roman villas such as Les Arenes and La Font del Vilar [32] (Fig 1A), and of centuriated grids spanning the northern littoral plain between the river Fluvià and the Albera Massif [88]. However, the relatively gradual reduction in oak woodlands, and low charcoal abundance over much of this phase, suggests that the impact of Roman occupation and land management was progressively increasing but not so extensive in coastal areas and woodlands. Pollen evidence from the Sobrestany [81] and Ullastret [89] lakes depict a similar context in the southern Empordà basin for the Roman period, with short-lived agropastoral openings occurring within an overall wooded coastal landscape. Higher grazing pressure is inferred in the EM core by increasing trends of dung-related fungi after 300 AD, reaching a maximum at ~500 AD. The re-occupation of the neighbouring site of Roses, at the ancient Greek colony of *Rhode* (Fig 1A), after the mid-2^nd^ century AD and its development into a prosperous commercial harbour city during Visigoth rule between the 5^th^ and 6^th^ centuries AD [33] most likely fostered the agropastoral exploitation of nearby wetlands.

### Phase IV [~750–~1250 cal AD]. *A closed lagoon*, *maximum landscape impact and clearance of coastal woodlands*

Geochemical and biotic indicators provide evidence for the culmination of the lagoon’s closure, which began in the previous phase. Firstly, a reduction in salinity from marine towards more brackish is suggested by the dominance of the ostracod assemblages by euryhaline *Cyprideis torosa* and brackish species *Loxoconcha elliptica*, with the reduction of more stenohaline brackish and marine species, and the appearance and maxima in taxa typical of temporary waters with variable salinity (*Sarcypridopsis aculeata*, *Herpetocypris* sp.) (Fig 5). Secondly, palynological data indicate the expansion of salt marshes (Fig 9). In the geochemical data, the the Sr/Ca ratio indicates that this process reached its maximum at ~ AD 1090. At this moment the freshwater and marine recharge through the inlets and sea connections was minimum, favouring water column reduction and higher nutrient loading, which in turn fostered euxinic bottom conditions. This is indicated by the presence of pyrite, the rise of P and a peak in the organic matter/S ratio (Fig 3), a process which has been observed in other lagoon environments [90]. The increase of S*piniferites* spp. cysts (Fig 8) could be also related to increased eutrophic conditions, as recorded in modern records from the Eastern Mediterranean [91]. The palaeoenvironmental data indicate closure of the Castelló lagoon during the mid-8th century, a process for which documentary sources do not exist. Later written sources do attest to the existence of the Castelló lagoon as “*Stagnum Castilionis*" in 944 AD [92], and toponymic data from the mid-10^th^ century record several saline wetlands–*Stagno Salatam* and *Stagno Sanguinario*- within the Castelló and nearby Sant Pere lagoons [93], which is in accord with our results.

During the mid-8^th^ century AD a major landscape opening is documented, with the large-scale and long-lasting clearance of littoral woodlands and the expansion of pasturelands (Figs 7 and 9). This included the clearance of cork oak woodlands growing in nearby ranges which, being replaced by tree heath (*Erica arborea*-type) and rockrose (*Cistus*) scrubland, would only partially recover in the following centuries. The concomitant increase in macrocharcoal suggests the use of fire for deforestation. This major change in land-use practices correlates with the Carolingian conquest and the pioneer settling of Christian monastic communities in the area. Archaeological and documentary data support the existence of monastic cells near the Castelló lagoon: Sant Pere de Rodes since the 8^th^ century [33], and Sant Quirze de Colera and Sant Joan Sescloses since the 9^th^ century AD [92, 94] (Fig 1B). The colonization of this frontier zone was incited by Carolingians through the “aprisió” landholding system, granting ownership of undeveloped lands to new settlers through their clearance and cultivation [95]. The implementation of this system largely impacted the Empordà basin by putting in place an overall open coastal landscape. This is confirmed regionally by the Sobrestany lagoon’s pollen record in the Baix Empordà, where oak woodlands were cleared sometime after the mid-7^th^ century AD [81]. However, oak stands remained untouched until the 11^th^ century AD at other nearby coastal locations like the Portlligat Bay (Cap de Creus) [96], stressing the complexity of landscape management in the region. Landscape opening and intensified land-use exploitation was not confined to the coastal zone but also involved neighbouring highland Pyrenean ranges, as evidenced by marked woodland clearances documented in the eastern Pyrenean fens after the 8^th^ and 9^th^ centuries cal BC [97, 98]. Reduced tree cover and increased human exploitation in both the Pyrenean ranges and the Empordà basin probably fostered increased sediment deposition in littoral areas, thus contributing to the culmination of the lagoon closure. Landscape clearance was followed by the marked expansion of pasturelands and farming activities since the 11^th^ century AD, as indicated by the inferred increase in local grazing activities, and in olive and hemp cultivation (Figs 7 and 9). This coincides with Castelló d’Empuries becoming the capital of the Empúries earldom and the beginning of feudalism [33]. We relate the increased use of littoral wetlands and grasslands for grazing to the local development of rural farms or “cortals”, which until the mid-14^th^ century were mainly oriented to grazing and which included enclosed pasturelands or “closes” [34]. Cortals are mentioned in local early 13^th^ century written sources, but no systematic analysis of documentary sources for the period before the 14^th^ century has been performed, and historians have indeed proposed an earlier development of these littoral farms [34]. Our results would argue in favour of this hypothesis, supporting the grazing exploitation of littoral grasslands and wetlands by “cortals” since the 11^th^ century. Concomitant to this increase in farming activities, a major geochemical anomaly with high concentrations of metals is observed. These metal anomalies may be the result of mining activities exploiting arsenopyrites outcrops in the neighboring Pyrenean ranges [99]. Written sources from the 10^th^ century document the existence of iron furnaces in the ranges bordering the Empordà plain, such as the Albayà farga located in the upper course of the Muga river [100]. Geochemical analyses performed in the Cadí range show the significance of such Pyrenean medieval mining, especially during the 11^th^ century [101].

### Phase V [1250 cal AD—1700 AD]. *Water management and drainage of a freshwater lagoon for industrial and agricultural activities*

At ~ 1250 AD, a major and unprecedented change in the lagoon water chemistry is attested by the marked shift to all freshwater biotic assemblages, the dominance of calcite precipitation and the increase in organic matter content. The absence of gypsum is consistent with the disappearance of euxinic conditions in the sediments. Cold freshwater ostracods occurring between ~ 1250 and 1515 cal AD could be related to the first cold episodes of the Little Ice Age: the Wolf and Spörer minimums, recorded at ~1305±70 and 1470±160 cal AD, respectively in solar activity terrestrial archives [102]. It is possible that the evolution of a more permanent fresh water basin allows the influence of climate change to be defined more easily since salinity fluctuations in coastal lagoons depend both on climate and shifting marine influence.

The Castelló lagoon had been fed naturally by minor fluvial discharges originating in the Rodes range and by a marine inlet known as *ipso Gradu* in written sources from the 10^th^ century AD [92, 103]. The Muga river mouth was located to the south of the study area (Fig 1A). The rapid shift to a new, freshwater drainage regime could be due to several factors. Increased storming and flooding, documented regionally in the Tech basin of the northern slope of the eastern Pyrenees during the late 13^th^ and 15^th^ centuries AD [104], could have contributed to a natural avulsion of the Muga river into the lagoon. However, we do not find evidence for continued avulsion processes in the sedimentological record during the Little Ice Age, as would have been expected in the case of climatic-driven events. Besides, historical records report frequent Muga floods only after the 15^th^ century AD [105]. An additional argument that would argue against a natural avulsion of the river is that the abrupt right-angle turn that the river needs to take in order to drain into the lagoon (Fig 1) could hardly be attributed to natural causes. Instead, we interpret this shift to a freshwater regime as indicative of artificial water management practices. Written sources from 1239 AD document the existence of a canal collecting water from the Muga river village towards Castelló d’Empuries. This canal, known as the *Rec Comptal*, fed the Castelló village’s mills, watered its surrounding orchards [35], and most likely functioned as the village´s sewage. The exact itinerary and drainage of this canal is scarcely known, but our data suggests that this channel actually drained the Muga into the lagoon, changing its water regime and increasing the water column. The existence of a freshwater inlet at this moment is also supported by the abundance of *Reimeria sinuata* and *Achnanthidium minutissimum*, freshwater diatoms that are typical of flowing well-oxygenated waters with mild nutrient enrichment [106]. Later channelling works built in the 1330s AD continued draining freshwater from the Muga river into the lagoon after feeding a new watermill network built in the immediacy of the Castelló village [35]. Such late-medieval canals could be thus considered as precursors of the Mugueta channel, depicted in 17^th^ and 18^th^ century local maps [19, 107].

The watermill industry in the Empordà plain was extensive during the 13^th^ and 14^th^ centuries AD, with more than 50 flour and fulling watermills documented along La Muga basin [35]. The channel conducted wastewater discharges from these watermills into the lagoon, after supplying water for the export-oriented Castelló village craft industry including a wide range of activities such as fulling, dying, tanning, bleaching, and grain gristing [108]. Wastewater generated by all these industrial activities contributed to an increased nutrient load in the lagoon. This is mainly evidenced by the increased organic matter and P content. The OM/S ratio shows an oxygenated sedimentary environment related to the lack of sulphates and the disappearance of pyrite. These phosphorus and nutrient-rich conditions favoured blooms of the algae *Gloeotrichia*-type [109], and the occasional presence of the eutrophic planktonic diatom *Cyclostephanos invisitatus* between 1250 and 1450 AD. The species composition of benthic diatoms and presence of charophyte gyrogonites suggest mesotrophic conditions and clear, shallow waters. The anthropogenic control of the lagoon’s drainage system may have favoured geomorphological bottom irregularities with standing water pools and flowing water inlets that allowed for diverse trophic conditions. The influence of such morphological irregularities in the trophic status of modern Mediterranean lagoons has been widely reported [110].

The construction of the channel contributed to the progressive infilling of the southwestern part of lagoon between the mid-13^th^ and 17^th^ centuries AD. This process is first attested by a rise in SARs and an inferred shallowing, as shown by the disappearance of hydrophytes and bulrush (*Typha*), non-siliceous algal remains and abundance peak of aerophilous diatom species such as *Luticola mutica* and *Hantzschia amphioxys* prior to their disappearance from ~1450 AD, indicating the start of a desiccation trend. Dried lands (“aiguallexos”) developed near the channel as a consequence of silt deposition creating land which was reclaimed for farming. The rise of dung-related fungi during this phase is consistent with the expansion of grazing in the reclaimed lands, particularly since the 14^th^ century AD, when different “cortals” are indeed recorded in late 14^th^ century sources only 1 km southwest of the EM site [34], and new “aigualleixos” appear after the early 17^th^ century to the south and west of the coring site [18]. A later stage of this progressive infilling is attested in the late 17^th^ century, when fluvial deposits overlapped the lagoon muds and the site was effectively dried. This resulted from the Muga avulsion joining the Mugueta channel into the lagoon, as depicted in ancient maps since ~1630 [107].

During this phase the brackish event observed at ~1560 AD could be related to a storm episode introducing marine waters in the lagoon. Evidence of a severe storm with strong winds and high waves is attested in 1552, when the Castelló lagoon significantly increased its perimeter to cover the distance between the Castelló and Roses villages [111].

## Conclusion

The multi-proxy analysis of the EM core combining geochemistry, mineralogy, ostracods, diatoms, pollen, NPPs and charcoal has provided key information for 1) reconstructing the transition of the Castelló lagoon from a marine to a marginal lagoonal environment through five evolutionary phases dated between 3150 cal BC to the 17^th^ century cal AD; 2) tracking saline fluctuations; 3) disentangling the natural and anthropogenic forces contributing to changes in littoral environments; and 4) documenting the exploitation and management of human societies of both the lagoon and its surrounding landscapes.

From the Late Neolithic to the Medieval period (3150 cal BC-750 cal AD) the lagoon ecosystem was mainly triggered by changing marine influence resulting from the successive development of littoral beach ridges and sea-level change. During this period human land-uses produced a limited impact on both the lagoon and its surrounding landscape dominated by oak woodlands. The diverse topography of the lagoonal system provided a range of habitats which were highly attractive for prehistoric societies, most significantly pasturelands that were grazed by Neolithic groups. However, this activity caused but moderated openings. In the same manner, agropastoral exploitation during the Iron Age and the Antiquity resulted either in short-live or moderate impacts within an overall wooded landscape. Also to be stressed for this period are human responses and land-use adaptation to natural coastal dynamics. This is evidenced between ~1550–~150 cal BC (Phase II), when maximum marine flooding hampered agropastoral exploitation of coastal wetlands near the EM site, being only briefly resumed during reduced marine influence between the 8th and 6th centuries cal BC.

In contrast, societies become a major agent of landscape transformation during the Medieval period, actively controlling the lagoon dynamics and resources. This is evidenced with the removal of littoral woodlands and the expansion of agrarian activities following the 8^th^ century Christian settlement in the region, factors that most likely contributed to the lagoon’s closure. The development of Pyrenean mining and smelting activities since the 11^th^ century polluted the Castelló lagoon with heavy metals. The flourishing of the late-Medieval textile and flour-milling industries in Castelló d’Empύries and the increased need for agricultural lands led to the ultimate channelization of the river Muga into the lagoon after 1250 cal AD. The anthropic control of the lagoon caused a profound impact in the lagoon dynamics including 1) its transformation into a freshwater lake, 2) increased nutrient load resulting from industrial activities, and 3) the ultimate infilling and drainage of a greater part of the Castelló lagoon for land reclamation, a process that affected our study location in the 17^th^ century AD.

This study stresses the value of lagoonal records for modelling the long-term relationship between highly diverse and dynamic littoral ecosystems and human resource exploitation. The Castelló lagoon record is important in that it tracks the previously undocumented shift from a naturally-driven to an anthropogenically-controlled system, from around 750 yr ago. Current and future management policies focusing on the conservation and restoration of Mediterranean lagoonal resources need to take into account that these are ancient and heavily-modified systems, with long-term anthropogenic impacts and controls that cover multi-centennial or even millennial timescales. While the widespread anthropogenic modification of lagoons is acknowledged in their classification as ‘artificially-modified’ in the European Union Water Framework Directive [112], our study demonstrates that, as in eutrophic shallow lakes across Europe [113] palaeoenvironmental data from lagoons are extremely important in attempting to define the natural ‘baseline’ state as the ideal restoration target. Multi-proxy palaeoenvironmental data and the construction of a reliable radiocarbon chronology allowed us to reconstruct the complexity of natural and anthropic processes affecting lagoonal ecosystems, which are either underestimated or are even not considered in written records. In this respect, our calibration of the nearest ΔR offset for this region will significantly contribute to the construction of reliable chronologies based on marine shells in the eastern Iberian Peninsula.

## Supporting Information

S1 Fig Stratigraphic comparison of zone boundaries defined by the different proxies. (TIF)

S2 Fig Elemental concentrations of studied bulk elements. (TIF)

S1 Table Location of cores obtained in this study (a-e) and previous existing cores (f). (DOCX)
