# Supplementary figures and images for: Coastal Evolution in a Mediterranean Microtidal Zone: Mid to Late Holocene Natural Dynamics and Human Management of the Castelló Lagoon, NE Spain
Source: PLoS One. 2016 May 13;11(5):e0155446. doi: 10.1371/journal.pone.0155446 (PMC4866732; doi:10.1371/journal.pone.0155446)

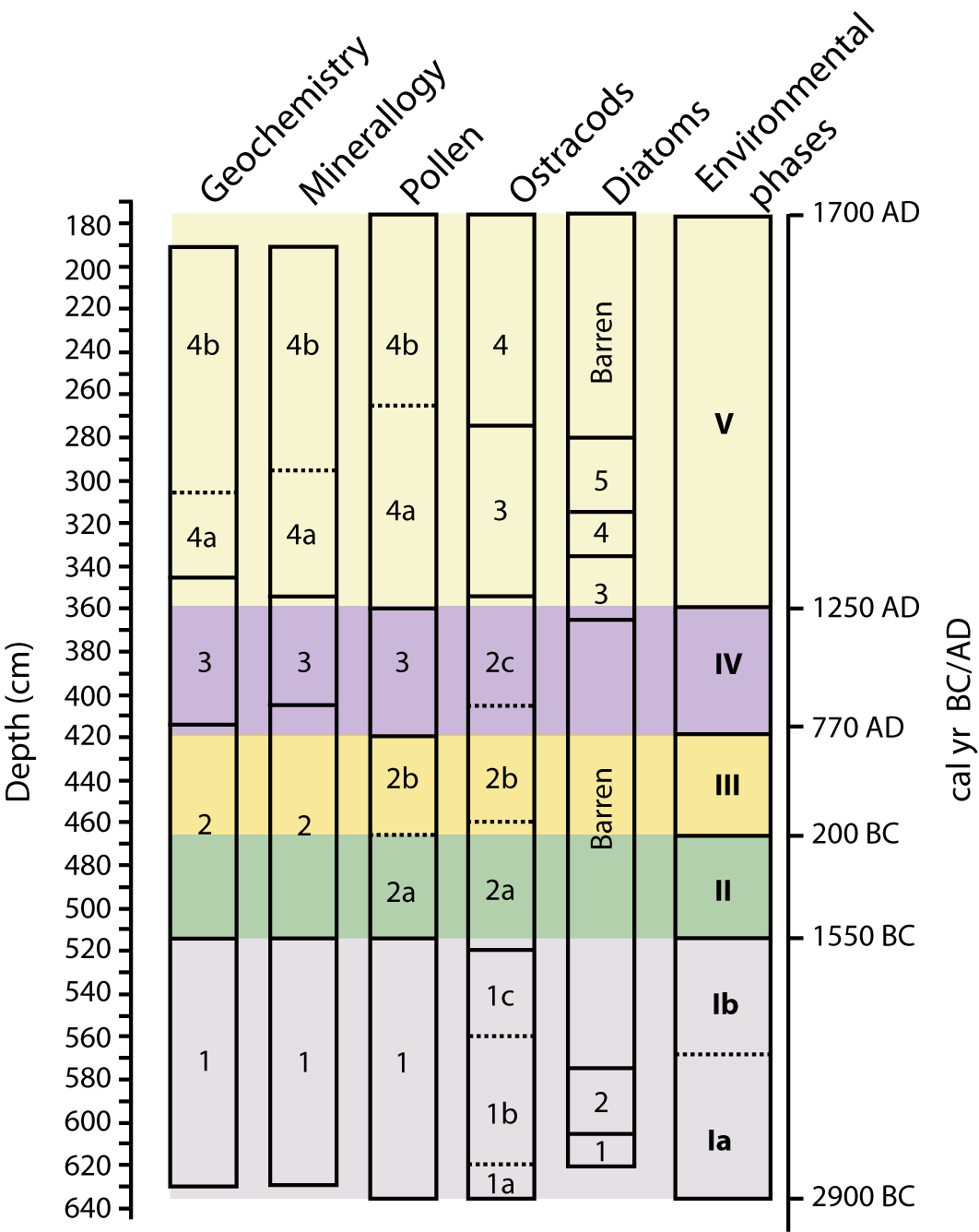

Supplement: S1 Fig — (TIF) [file pone.0155446.s001.tif]

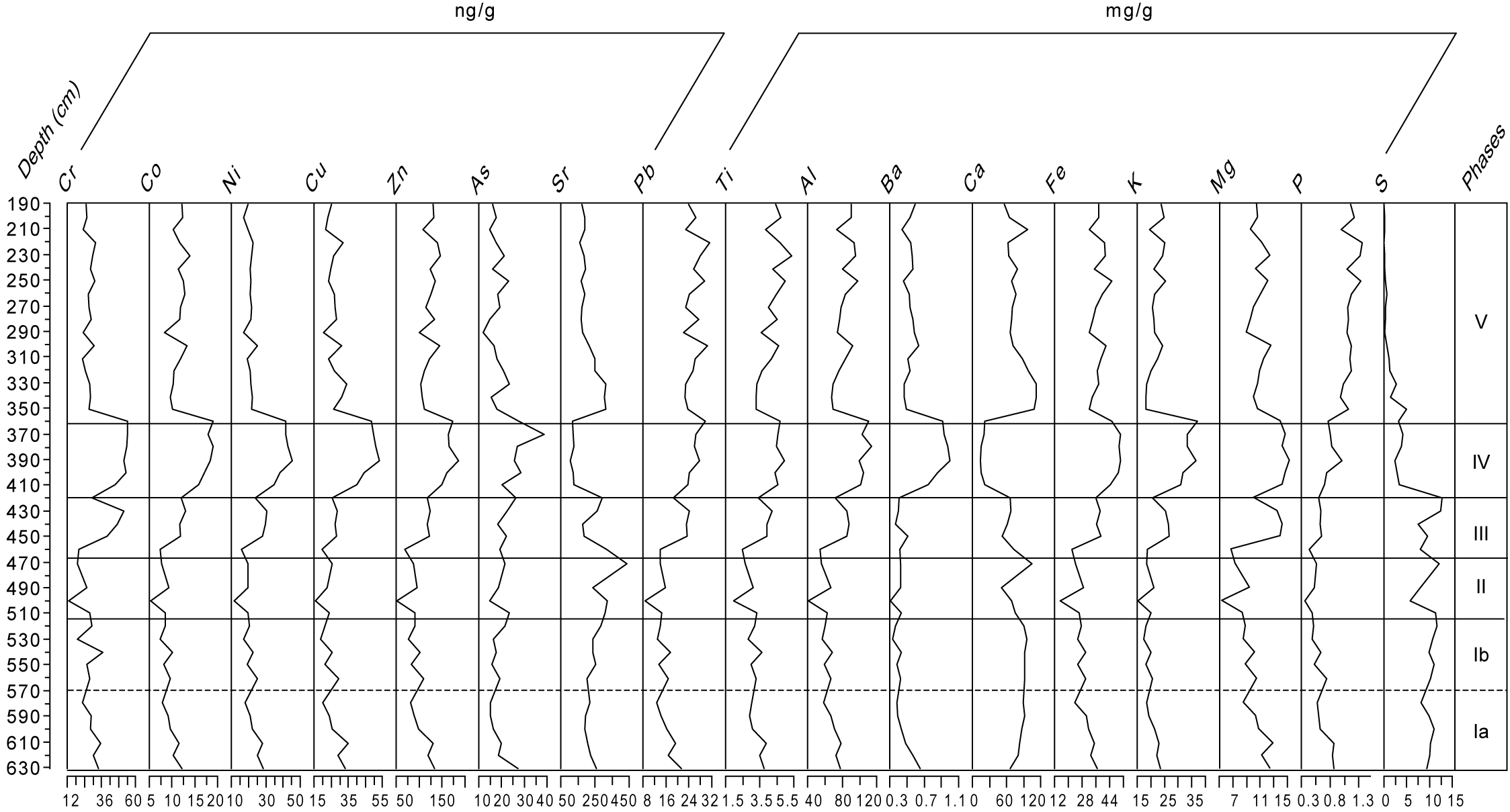

Supplement: S2 Fig — (TIF) [file pone.0155446.s002.tif]
