# Supplementary material for: Coastal Evolution in a Mediterranean Microtidal Zone: Mid to Late Holocene Natural Dynamics and Human Management of the Castelló Lagoon, NE Spain
Source: PLoS One. 2016 May 13;11(5):e0155446. doi: 10.1371/journal.pone.0155446 (PMC4866732; doi:10.1371/journal.pone.0155446)
Supplement: S1 Table — (DOCX) [file pone.0155446.s003.docx]

| **Core site** | **Name** | **Latitude** | **Longitude** | **Altitude (m a.s.l)** |
| --- | --- | --- | --- | --- |
| a | Closa Ullal Montmajor | 42ᴼ 17’ 23’’ N | 03ᴼ 05’ 22’’ E | 2.2 |
| b | Estany de Pau | 42ᴼ 17’ 27’’ N | 03ᴼ 05’ 41’’ E | 1.4 |
| c | Estany d’en Mornau | 42ᴼ 16’ 54’’ N | 03ᴼ 05’ 58’’ E | 2.4 |
| d | Closes del Tec | 42ᴼ 16’ 33’’ N | 03ᴼ 07’ 11’’ E | 1.9 |
| e | Vila | 42ᴼ 16’ 11’’ N | 03ᴼ 07’ 20’’ E | 1.4 |
| f | Estanys de Palau | 42ᴼ 16’ 53’’ N | 03ᴼ 07’ 13’’ E | 1.4 |
